# Innovative Biomaterials for the Treatment of Bone Cancer

**DOI:** 10.3390/ijms22158214

**Published:** 2021-07-30

**Authors:** Luca Ambrosio, Maria Grazia Raucci, Gianluca Vadalà, Luigi Ambrosio, Rocco Papalia, Vincenzo Denaro

**Affiliations:** 1 Laboratory of Regenerative Orthopaedics, Department of Orthopaedic and Trauma Surgery, Campus Bio-Medico, University of Rome, Via Alvaro del Portillo 200, 00128 Rome, Italy; g.vadala@unicampus.it (G.V.); r.papalia@unicampus.it (R.P.); denaro@unicampus.it (V.D.); 2 Institute of Polymers, Composites and Biomaterials, National Research Council (IPCB-CNR), Viale J.F. Kennedy 54, Mostra d’Oltremare Pad. 20, 80125 Naples, Italy; mariagrazia.raucci@cnr.it (M.G.R.); luigi.ambrosio@cnr.it (L.A.)

**Keywords:** bone metastasis, osteosarcoma, biomaterials, nanocarriers, musculoskeletal oncology, tissue engineering, bone regeneration

## Abstract

Bone cancer is a demanding challenge for contemporary medicine due to its high frequency of presentation and significant heterogeneity of malignant lesions developing within the bone. To date, available treatments are rarely curative and are primarily aimed at prolonging patients’ survival and ameliorating their quality of life. Furthermore, both pharmacological and surgical therapies are aggravated by a consistent burden of adverse events and subsequent disability due to the loss of healthy bone structural and functional properties. Therefore, great research efforts are being made to develop innovative biomaterials able to selectively inhibit bone cancer progression while reducing the loss of bone structural properties secondary to local tissue invasion. In this review, we describe the state of the art of innovative biomaterials for the treatment of bone cancer. Along with physiological bone remodeling, the development of bone metastasis and osteosarcoma will be depicted. Subsequently, recent advances on nanocarrier-based drug delivery systems, as well as the application of novel, multifunctional biomaterials for the treatment of bone cancer will be discussed. Eventually, actual limitations and promising future perspectives regarding the employment of such approaches in the clinical scenario will be debated.

## 1. Introduction

According to the World Health Organization (WHO), cancer is the second leading cause of death worldwide, accounting for nearly 1 out of 6 deaths [1]. The high mortality associated with malignant tumors is primarily due to the dissemination of metastatic cells to distant sites, thus causing multiple organ dysfunction, chronic pain, and eventually death [2,3]. Together with the liver and lung, bone is the most common site of metastasis, especially in patients affected by advanced-stage carcinomas of the prostate, breast, and lung [4]. This is particularly crucial as such malignancies are the most common primary cancers globally, with breast cancer accounting for the highest number of new cases in 2020 [5]. It has been estimated that up to 75% of patients affected by stage IV breast cancer will develop a bone metastasis [6], while approximately 20% of patients with early-stage breast cancer will present bone metastatic lesions after a mean follow-up of 8.4 years, according to a large cohort study [7]. Furthermore, bone metastases may be already evident in up to 47.7% of patients at the initial diagnosis, with a subsequent median overall survival of 40 months [8].

Bone metastases often result in skeletal-related events (SREs) including uncontrolled pain, pathological fractures, hypercalcemia, and spinal cord compression, which may further impact the quality of life of patients with advanced-stage tumors and a poor prognosis [9]. Treatment of SREs is multimodal and frequently involves the administration of drugs (i.e., analgesics, bisphosphonates, RANKL inhibitors, radiopharmaceuticals) as well as locoregional (i.e., radiotherapy, vertebroplasty, kyphoplasty) and surgical approaches (i.e., nerve decompression and/or fracture fixation surgeries). However, these treatments mainly aim at decreasing the rate of SREs and only a small fraction of them can transiently reduce metastatic cell turnover and proliferation, thus achieving local tumor control [10]. In addition, they are often aggravated by serious side effects (e.g., hypocalcemia, kidney failure, osteonecrosis of the jaw for most anti-resorptive drugs [10], myelotoxicity and a higher risk of fracture following radiation therapy [11]) that may further increase the disease burden in treated patients.

Although significantly less common (accounting for approximately 0.2% of all malignant tumors), primary bone cancers are characterized by considerable morbidity and mortality. Nearly 70% of all cases are represented by osteosarcomas, which present a yearly incidence of approximately 1–3 in 1 million cases and mostly affect children and adolescents, with a second peak in patients aged over 50 [12]. Classically, wide or radical resection of the entire tumor-bearing compartment (often involving a whole limb) has been considered as the gold standard treatment for musculoskeletal solid cancers, eventually resulting in highly invasive and mutilating operations with a significant burden of complications. However, the introduction of innovative multimodal treatments has reduced the aggressivity of traditional surgical approaches while being equally effective on local and systemic recurrence and disease-free survival [13].

Therefore, great research efforts are being made toward the development of novel treatments that may inhibit both primary and secondary bone cancer progression while reducing the loss of bone structural properties as a consequence of local tissue invasion.

In the last decade, several biomaterials have been proposed as innovative solutions for the local delivery of chemotherapeutics (e.g., polyethylene glycol (PEG), polyethylene oxide, poly ε caprolactone, polyvinyl alcohol (PVA), polymethyl methacrylate (PMMA), chitosan (CS), alginate, cellulose, etc.), bone fillers, magnetite-based materials for hyperthermia treatment as well as smart multifunctional materials provided with both structural and therapeutical properties [14].

The purpose of this review is to describe the current state of the art on the development of innovative biomaterials to treat metastases and primary cancers of the bone. Biological mechanisms underlying cancer onset and development within the bone will be elucidated as well as main molecular players involved in the process. Subsequently, a description of cutting-edge systems and materials under investigation will contextualize their role in the treatment of bone cancer. Finally, future perspectives and actual limitations of biomaterials in current use will be discussed.

## 2. Bone Cancer Development and Progression

### 2.1. Bone Physiology and Turnover

Bone is a dynamic organ constantly undergoing structural remodeling, according to the needs of local tissue homeostasis. The main players involved in this process are bone-forming cells, namely osteoblasts, which belong to the mesenchymal lineage and are capable of synthesizing the mineralized bone extracellular matrix (ECM) and, on the other front, osteoclasts, which derive from hematopoietic macrophage/monocyte precursors and are responsible for bone matrix resorption. The balance toward either process (e.g., bone formation or resorption) is dictated by a multitude of factors, including both endocrine and paracrine mediators as well as physical stimuli [15,16].

Osteogenic mediators such as vitamin D_3_, bone morphogenetic proteins (BMPs), endotelin-1 (ET-1), platelet-derived growth factor (PDGF), transforming growth factor-β (TGF-β), fibroblast growth factor (FGF), insulin-like growth factor-1 (IGF-1), epidermal growth factor receptor (EGFR) and growth hormone (GH) stimulate the differentiation of bone marrow mesenchymal stem cells (BM-MSCs) toward the osteoblastic lineage and the synthesis of bone matrix through the activation of the transcription factor Runx-2 [17], as well as osteoblast proliferation and survival [15]. Following the deposition of newly secreted matrix, osteoblasts become entrapped in lacunae, eventually transitioning to osteocytes, stellate-shaped cells communicating via cytoplasmic processes extending within bone canaliculi [18]. Through this vast network, osteocytes are able to sense mechanical and biological stimuli from the surrounding microenvironment and modulate both osteoblast and osteoclast activity [19].

On the other hand, osteoclast differentiation and recruitment are promoted by local factors including interleukin (IL)-1, IL-6, and macrophage colony-stimulating factor (MCSF), while they are inhibited by systemic mediators such as calcitonin [15]. The receptor activator of nuclear factor-κB (NF-κB) ligand (RANKL)/RANK/osteoprotegerin (OPG) axis plays a fundamental role in osteoclastogenesis and osteoclast activation. RANKL is a member of the tumor necrosis factor (TNF) family and is released by osteoblasts and T-cells in response to pro-osteoclastogenic stimuli. Once RANKL interacts with its receptor (RANK) expressed by osteoclast precursors, these cells are activated, thence resulting in bone resorption. OPG, a soluble decoy receptor for RANKL, is physiologically secreted by osteoblasts to limit excessive osteoclast activity via inhibiting RANK/RANKL interaction, thus suppressing osteoclastogenesis and bone resorption [20]. In addition, OPG is also a decoy receptor for TNF-related apoptosis-inducing ligand (TRAIL), which is able to induce apoptosis in mature osteoclasts [21]. As OPG has an equally high affinity for both RANKL and TRAIL, it is simultaneously capable of reducing osteoclast activation and apoptosis [22].

Bone remodeling is also regulated by several systemic mediators and hormones which convey on different pathways both on bone formation and resorption [15]. Parathyroid hormone (PTH) regulates bone mass in an endocrine manner and can either foster catabolic or anabolic effects on bone. Low and intermittent doses of PTH increase bone formation both directly (through the stimulation of osteoblast proliferation, differentiation, and survival) and indirectly (via increasing the release of IGF-1) [23,24], while continuous administration of PTH promotes bone resorption via stimulation of RANKL and inhibition of OPG expression [15]. PTH-related peptide (PTHrP) shares a relevant part of its biologically active domain with PTH, is secreted in almost all tissues, and acts in an autocrine/paracrine manner. It has been demonstrated to hold an important role during the embryonic development of the skeleton [25] as well as regulating postnatal bone formation through the interaction with the PTH receptor [26].

Estrogen is one of the major regulators of bone remodeling in both men and women. Indeed, it inhibits osteoclast activity via decreasing RANKL expression as well as the production of osteoclastogenic cytokines (IL-1, IL-6, TNF-α, MCSF, and prostaglandins) [15] while stimulating osteoblast survival and anabolism [27].

### 2.2. Development and Progression of Bone Metastases

According to the classic “seed and soil” theory proposed by Paget in 1889, cancer cells tend to metastasize toward a favorable microenvironment [28]. More commonly, metastases are localized in bones with a high degree of vascularization, trabecular bone content, and red marrow activity, such as the vertebrae, pelvis, ribs, and meta-epiphyseal regions of long bones [29]. Indeed, the bone marrow represents a metabolically active florid niche enriched with growth factors and specific biological cues that facilitate tumor cell homing into the bone [30]. This process is fostered by the overexpression of specific chemokine receptors in bone-homing tumor cells, namely C-X-C motif chemokine receptor 4 (CXCR-4), which is normally expressed in hematopoietic stem cells (HSCs). Its ligand, C-X-C motif chemokine ligand 12 (CXCL-12), is constitutively produced by osteoblasts, fibroblasts, and endothelial cells to retain HSCs within the bone marrow, thus facilitating cancer cells aberrantly overexpressing CXCR-4 to migrate into the bone [31,32]. Other chemokine axes, such as CXCR-6/CXCL-16 and CXCR-3/CXCL-10, seem to be involved in this process and may thus be considered as promising immune checkpoints for targeted cancer therapy [33]. Furthermore, breast, prostate and kidney cancer cells often overexpress calcium-sensing receptors (CaSR), which promote migration toward Ca^2+^-rich tissues such as the bone as well the release of growth factors, proangiogenic factors, and chemokines that support proliferation and invasion of metastatic cells [34]. In addition, CaSR stimulation in cancer cells upregulates the synthesis of PTHrP thence promoting osteoclastic activity via increasing the expression of RANKL and decreasing the release of OPG [35]. Increased bone resorption contributes to the development of metastatic osteolytic lesions and the release of bone-derived growth factors, which may further support the survival and proliferation of cancer cells [36]. This process has been defined by Mundy et al. as the “vicious cycle” of bone metastasis self-maintenance and progression [37]. Additionally, PTHrP-related excessive release of extracellular Ca^2+^ may be associated with the onset of humoral hypercalcemia of malignancy, a common paraneoplastic syndrome found in advanced-stage breast, prostate, and kidney cancer as well as in multiple myeloma [38]. Differently, if bone formation prevails over resorption, a sclerotic lesion may develop. It is hypothesized that the release of growth factors from cancer cells (e.g., TGF-β, FGF, BMPs, Wnt) may enhance osteoblast differentiation, proliferation, and metabolism while inhibiting osteoclast activity [10]. However, it is widely accepted that both mechanisms, namely lytic and sclerotic, commonly overlap during metastasis development and progression [39]. Together with metabolically active cells, a variable quantity of metastatic cells enters a state of dormancy in which they tend to acquire osteoblast and osteoclast surface markers thus becoming able to escape the immune response and anticancer drugs [40]. Based on cues emanating from the bone microenvironment, such condition may indefinitely persist or transition to cell activation and metastasis progression [41].

### 2.3. Biological and Molecular Characteristics of Osteosarcoma

Osteosarcoma mainly occurs in long bones (femur, tibia, humerus), in the proximity of the metaphyseal growth plate, characterized by high metabolic activity and cell turnover [42]. This cancer commonly shows an aggressive clinical course, with the propensity to destroy and invade local tissue as well as metastasize to the lungs and other sites [43]. Current conventional treatment mainly involves a neoadjuvant scheme based on a combination of cisplatin (CDDP), doxorubicin (DOX), methotrexate (MTX) and/or other drugs, followed by limb-sparing or amputation surgery [44,45]. However, about 30% of patients will not respond to the treatment and only 20% will survive more than 5 years with relapsing or metastatic osteosarcoma [42]. The inefficacy of therapy may be partly explained by the high degree of heterogeneity both within the tumor itself and among diverse individuals. Indeed, cancer cells may be characterized by different degrees of chromosomal aneuploidy, genomic instability, and number of genetic mutations, hence altering their response to therapeutics [46].

Osteosarcomas are primarily constituted by transformed osteoblastic cells producing osteoid matrix, probably derived by MSCs and/or pre-osteoblasts undergoing p53 and Rb pathway disruption, along with other recurrent genetic mutations [47]. Consequently, these rapidly proliferating cells aberrantly alter the dynamics of bone remodeling leading to the development of aggressive, osteolytic lesions causing bone fragility and increased risk of pathologic fractures. Furthermore, the degree of osteolytic resorption is also correlated with a poor outcome [48]. More specifically, bone resorption in osteosarcoma due to increased osteoclastic activity associated with aberrant RANK/RANKL signaling leads to the release of growth factors from the bone matrix, such as IGF-1 and TGF-β [49]. In particular, the latter seems to be implicated in both primary tumor growth and metastatic progression [50]. In addition, recent studies have demonstrated that extracellular vesicles (EVs) secreted by osteosarcoma cells are capable of promoting osteolysis, while RANK-bearing EVs released by osteoclasts may, in turn, activate RANKL expressed on cancer cells, thence resulting in osteolysis [51].

Additionally, osteosarcoma cells are also able to influence MSC activity and metabolism. Indeed, it has been shown that, when in contact with osteosarcoma cells, MSCs may acquire a cancer-associated fibroblastic phenotype and promote cancer cell motility, invasiveness, and transendothelial migration [52]. Moreover, it has been described that MSCs are able to support osteosarcoma cell survival and migration via a cargo of metabolites packed in EVs released from MSCs and subsequently uptaken by cancer cells [53]. On the other hand, osteosarcoma cells may release EVs rich in matrix metalloproteinase (MMP)-1, vascular endothelial growth factor (VEGF), and adhesion molecules, which favor bone remodeling, neoangiogenesis, and metastasis development [54].

## 3. Innovative Biomaterials for the Treatment of Bone Cancer

### 3.1. Inorganic Drug Delivery Systems

Current standard treatments for bone cancer often involve strategies aimed at achieving local tumor control (i.e., surgical resection, radiotherapy) followed by pharmacological treatments. However, these treatments are not able to effectively destroy cancer cells and do not substantially improve patients’ quality of life [55]. Nanomedicine may be a potential tool to achieve this aim. Recent studies focused on precision medicine have described the efficient delivery of antitumor therapeutics to bone cancer sites [56]. As drug delivery nanocarriers for anticancer treatment, great attention is paid to systems based on inorganic and/or organic materials. The advantages of these systems are mainly due to the improvement of bioavailability, pharmacokinetics, and tailored release of a loaded anticancer agent in the tumor site, thus determining an increase of therapeutic efficiency of the delivered drugs [57]. Here, calcium phosphate (CaP), calcium carbonate (CaCO_3_), mesoporous silica (MS), and cerium oxide (CeO_2_) are reported among the most used inorganic materials for the production of delivery systems.

CaCO_3_ in the form of micro- and nanoparticles or microspheres is widely used in drug delivery for the treatment of many types of tumors (i.e., osteosarcoma) for its important physicochemical and biological properties such as slow biodegradation, pH-sensitivity, good biocompatibility, osteoconductivity, and tailored drug release [58,59,60,61].

However, several studies reported the use of hybrid materials based on the combination of an inorganic component represented by CaCO_3_ and an organic phase because of their multiple advantages [59]. For example, Mao et al. [62] prepared micelles based on CaCO_3_-crosslinked methoxy poly(ethylene glycol)-block-poly (L-glutamic acid) (mPEG-b-PGA) for the release of MCSF, which is able to inhibit tumor growth by activating the immune response. In addition, Ding and colleagues produced DOX-loaded hyaluronate-CaCO_3_ hybrid nanoparticles able to promptly release DOX in the acidic tumor microenvironment, with significant anticancer efficacy [63]. Furthermore, among hybrid nanomaterials, the CaP/CaCO_3_ mineralization of polymers represents an important strategy for drug delivery system development for their remarkable advantages, such as: (i) the nanosystem is stable at physiological conditions while becoming sensitive at the acidic pH value typical of the tumor microenvironment, thus releasing Ca^2+^ ions and CO_2_ in the microenvironment [64]; (ii) the nanosystem shows good biocompatibility and biodegradability and may be easily eliminated by physiological processes [65]. In particular, Li et al. [66] developed CaCO_3_-core crosslinked nanoparticles of methoxy poly(ethylene glycol)-block-poly(L-glutamic acid) through a mineralization strategy for the release of DOX in a cell microenvironment with higher loading capacity and anticancer activity both on in vitro and in vivo osteosarcoma models. It is well known that the tumor microenvironment is characterized by a high level of reactive oxygen species (ROS) and many studies have reported the possibility to reduce the tumor bulk through up- or down-regulation of ROS. Among inorganic materials, CeO_2_ shows a dual ability to scavenge and generate ROS according to the change of pH value at the intra- and extracellular level. Indeed, CeO_2_ has the ability to scavenge ROS at physiological pH (7.4), while it generates ROS at acidic pH (6.0), a typical value of the tumor microenvironment. This ability allows obtaining a selective effect of CeO_2_ nanoparticles on healthy and cancer cells. Therefore, many studies reported their use with or without the combination with other treatments [67,68]. Furthermore, Tapeinos et al. [69] demonstrated that the delivery system developed by loading CeO_2_ nanoparticles (<25 nm) and DOX in CaCO_3_/collagen type I composite material was able to lead to about 100% osteosarcoma (Saos-2) cell death at pH 6.0, due to the synergy between the pro-oxidant effect of CeO_2_ and anticancer effect of DOX.

Mesoporous silica nanoparticles (MSN) are used as an anticancer drug delivery system for their favorable properties such as high surface area, biocompatibility, and easy functionalization. The porous structure allows the loading of drugs and can be functionalized with other molecules. To enhance the release efficacy, the gate of the pore can be opened once it reaches the target site through a specific stimulus (i.e., temperature, enzyme, light, pH). pH-responsive MSNs are one of the potential carriers to release anticancer drugs to the targeted site [70,71]. Sun et al. [72] developed gold nanoparticles enclosed in MSNs (Au@MSNs) and conjugated with zoledronic acid (ZOL) for the treatment of breast cancer bone metastases. They reported that, by combining loaded Au@MSN and photothermal treatment, both inhibition of tumor growth by inducing apoptosis and the improvement of bone microenvironment were obtained. Vallet-Regì et al. [73] developed an innovative nanodevice based on DOX-loaded MSNs nanoplatforms, where (1) polyacrylic acid was used as a core shell to prevent the fast release and provide the pH-responsive capacity to the system; (2) a targeting ligand, namely the plant lectin concanavalin A (ConA), was used to improve the selective action on cancer cells whilst preserving the viability of healthy cells. In vitro results demonstrated that only a small amount of DOX was needed to inhibit osteosarcoma cell proliferation, while the viability of healthy cells was preserved. Furthermore, the anticancer effect was increased up to 8-fold compared to the free drug.

CaP-based materials are inorganic biomaterials with good biological properties in terms of compatibility, bioactivity, and osteoinductivity with the capability to induce the osteogenic differentiation of MSCs and pre-osteoblast cell lines toward a mature osteoblast phenotype by increasing the gene expression of specific markers (i.e., alkaline phosphatase (ALP), osteopontin (OPN), osteocalcin (OCN), etc.), which could enhance osteointegration [74,75,76]. Among CaP materials, amorphous calcium phosphate (ACP) may be biodegraded by cells due to its unique structure, making it an ideal candidate as a delivery system for post-operative chemotherapy and to repair bone defects [77].

Zhou et al. [78] prepared microspherical structures based on calcium phosphate-phosphorylated adenosine (CPPA) by using a fast microwave-assisted solvothermal method (110 °C, 10 min). The CPPA system with the porous and hollow structure showed a high encapsulation efficiency of DOX (c.a. 42.3%) and displayed pH-responsive drug release properties. The loaded CPPA system showed a therapeutic effect on osteosarcoma cells both in vitro and in vivo. Furthermore, the CPPA system stimulates osteogenesis of human BM-MSCs, by expression of specific markers, such as ALP activity, OCN, OPN, and collagen type I.

In addition, Liu et al. [79] developed CaP with a porous microsphere structure at room temperature by using fructose-1,6-bisphosphate as a precursor of phosphorus. The authors demonstrated that loaded CaP microspheres showed a higher drug loading capability than the control represented by hydroxyapatite nanorods. Furthermore, the microspheres showed the best behavior in terms of cancer cell vitality inhibition and induction of osteogenic differentiation in MC3T3-E1 pre-osteoblasts. The results demonstrated the potential use of CaP as a drug delivery system and osteoinductive material for bone regeneration in the treatment of osteosarcoma [79]. A summary of the studies on inorganic drug delivery systems for the treatment of bone cancer is depicted in Table 1.

### 3.2. Polymeric Drug Delivery Systems for Bone Cancer Treatment

In recent years, great interest is raising toward the use of polymer-based anticancer drug delivery systems. In particular, injectable hydrogels have attracted considerable interest for the development of localized delivery platforms due to their important advantages, including application via minimally invasive approaches, appropriate biodegradability and a sustained drug release. Indeed, in situ administration of chemotherapeutic agents (i.e., DOX) by using hydrogel delays the local retention of the drug compared to systemic administration. Simultaneously, this approach significantly reduces the exposure of chemotherapeutic agents in healthy tissues compared to the free drug [80].

Dass et al. [81] have investigated the possibility to enhance anticancer activity through the co-delivery of DOX and anticancer genes by thermosensitive hydrogel systems. In particular, thermosensitive injectable hydrogels showed the ability to exist in the liquid state at room temperature and to solidify at a physiological temperature (37 °C). This behavior allows to easily mix the drugs in the polymer solution at lower temperatures, while drug-loaded hydrogels can be assembled soon after in vivo injection.

Instead, Ma et al. [82] have realized a tri-block thermosensitive poly(lactic-co-glycolic acid) (PLGA)−PEG−PLGA hydrogel loading three different drugs such as DOX, CDDP, and MTX in order to obtain a localized co-delivery of drugs. This system allowed to obtain a synergistic effect of drugs on in vitro osteosarcoma cell cultures (i.e., Saos-2, MG-63). Furthermore, on an in vivo osteosarcoma xenograft model, the co-release of anticancer drugs determined the highest tumor inhibition efficacy after 16 days with improved tumor apoptosis and increased regulation of the expressions of apoptosis-related genes.

Furthermore, several natural polymers have been used for designing an efficient drug release system for osteosarcoma treatment. Suksiriworapong et al. [83] reported a system consisting of MTX linked with poly(glycerol adipate) (PGA) which may self-organize in nanoparticles that showed good physical stability at pH 5–9 and ionic strength up to 0.15 M NaCl. Furthermore, the nanoparticles showed chemical stability against the hydrolytic reaction at physiological conditions up to 30 days but may be degraded by enzymatic cleavage thus allowing MTX release. Moreover, some studies reported that neat CS and its combination with other compounds, in different forms such as nanoparticles, scaffolds, and hydrogels, are efficient against cancer cells by inhibiting cell viability, increasing oxidative stress, and inducing cell apoptosis [84]. Ai et al. [85] investigated that copper-loaded chitosan nanoparticles (CuCNPs) showed higher anticancer activity than free copper sulphate used as control. The presence of functional groups of chitosan (-NH_2_ or -OH) allows obtaining a high loading of copper with a high stability. The nanoparticle size was <200 nm which is suitable for cancer treatment. The authors demonstrated that the anticancer activity was associated with a higher ROS production with higher caspase-3 and -7 activity. Furthermore, it was demonstrated that there was no difference in anticancer activity for either low or high molecular weight CS. Indeed, CS at both molecular weights inhibited Saos-2 cell viability and proliferation without significant difference [86]. The anticancer activity against osteosarcoma cells (i.e., MG-63) was also investigated by using a biomimetic scaffold based on CS-sodium alginate loading TiO_2_ nanoparticles [87]. In this case, TiO_2_ nanoparticles induced cell apoptosis through the endocytic pathway and involve superoxide dismutase (SOD1-2) activity and ROS production [87]. Other drug-delivery systems are based on the use of zeolites to entrap and modulate the release of loaded drugs. For example, Yang et al. [88] have produced nanodisks based on ZSM-5 zeolites core and a CS shell, which have been able to modulate the release of DOX to osteosarcoma cells. In this system, DOX was internalized in cancer cells by endocytosis and induced apoptosis.

In situ release was also obtained by using hyaluronic (HA) acid as an attractive ligand for targeted drug delivery to CD44-overexpressing cells, and HA modification could enhance the internalization of the nanocarrier in tumor cells [89,90]. Zhang et al. [91] have tested HA to develop a self-stabilized delivery nanogel. In particular, CDDP-crosslinked HA nanogel were synthesized to release DOX for the treatment of osteosarcoma. In this case, CDDP showed a dual function as both anticancer drug and crosslinked agent to inhibit the burst release of DOX and to achieve a synergistic therapeutic effect [91].

Moreover, hybrid nanoparticles based on an HA-PEG polymer shell and nano-hydroxyapatite core were developed to be loaded with ZOL. These hybrid nanoparticles showed high efficiency for drug loading, tailored drug release, and great biocompatibility. Furthermore, in vitro and in vivo investigations demonstrated the treatment at a low dose of nanoparticles allowed to obtain low cytotoxicity and tolerable immune response, demonstrating the potential application for bone cancer treatment [92]. Recent approaches have evaluated the use of immunochemotherapy for the treatment of osteosarcoma. Zhang et al. [93] have synthesized HA nanoparticles for tumor-targeted delivery of DOX, CDDP, and resiquimod (R848) in order to induce cancer cell apoptosis due to the effects of DOX and CDDP and, at the same time, to facilitate the tumor-antigen presentation and antitumor immunity induction due to the presence of R848.

**Figure 1:**
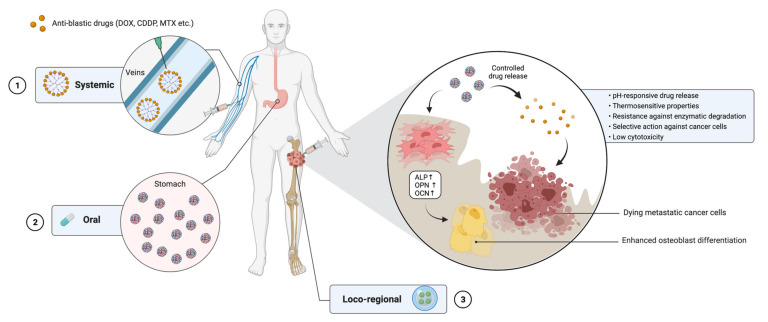
The use of nanocarrier-based drug delivery systems for the treatment of bone cancer. Nanocarriers loaded with chemotherapeutic agents may be administered either systemically (1), orally (2) or through a minimally invasive loco-regional approach (3). Once at the cancer site, nanocarriers are able to release the drug under different stimuli from the microenvironment and to selectively target cancer cells. On the other hand, biomaterials constituting the systems are able to increase MSC differentiation toward osteoblasts by upregulating the expression of target genes. DOX = doxorubicin; CDDP = cisplatin; MTX = methotrexate; ALP = alkaline phosphatase; OPN = osteopontin; OCN = osteocalcin. Created with BioRender.com on 18 May 2021.

The main characteristics of nanocarrier-based drug delivery systems are summarized in Figure 1. A summary of the studies on polymeric drug delivery systems for the treatment of bone cancer is depicted in Table 2.

### 3.3. Innovative and Multifunctional Strategies for Bone Cancer Treatment and Bone Regeneration

Currently, an innovative bone cancer therapeutic approach is based on photothermal treatment (PTT) as a minimally invasive procedure that can apply selective cytotoxicity on cancer cells, thus reducing detrimental effects toward healthy tissues. Moreover, many studies reported the effects of PTT on bone regeneration by enhancing cell proliferation (i.e., human mesenchymal stem cells, hMSCs) and inducing osteogenic differentiation [94,95]. To enhance PTT effects on bone cancer and bone regeneration, some transducing agents have been developed. Here, therapeutic biomaterials such as exfoliated black phosphorus, graphene oxide (GO), and carbon dots (CDs) for osteosarcoma treatment, are reported.

Fiorillo et al. [96] demonstrated that GO, prepared by the Hummers method and dispersed in a 5% mixture of dimethyl sulfoxide (DMSO) in double-distilled water, inhibits the formation of tumor-sphere in many cancer cell lines by blocking different signal transduction pathways such as WNT and Notch. Furthermore, it was reported that big GO sheets (5–20 µm) perform their effects by inactivation of signal transduction pathways which are initiated at the cell surface. Moreover, Raucci et al. [97] showed that GO, obtained from graphene nanoplatelets (GNPs) using a simplified Hummers method following the procedure reported in a previous study, is non-toxic for hMSCs and promotes their differentiation toward the osteoblast phenotype in a basal medium without osteogenic factors and PTT. Indeed, the possibility to combine the osteoinductive properties of calcium phosphate and GO by developing injectable GO-hydroxyapatite hybrid materials using the sol-gel approach has been demonstrated. Recently, Zhang and Ma [98] fabricated graphene nanoplatelets intercalated in gelatin/hydroxyapatite porous scaffolds with photothermal properties by bio-printing technology. The detailed composition of slurry for printing the porous structure and the specific parameters used during the process are reported in a previous work [98]. They found that the scaffold containing 0.5% graphene achieved the temperature of c.a. 43 °C in a few minutes (3 min) and may be restored after cooling for 10 min. The authors found that these scaffolds induced a significant increase in cell proliferation after 5 days of culture and 3 cycles of PTT at 40–43 °C. Furthermore, a multifunctional composite scaffold based on GO and β-tricalcium phosphate (GO/β-TCP) with photothermal properties was developed via 3D printing technology. The inks were obtained by mixing β-TCP powders in polyvinyl acetate solution (6 wt%). Then, scaffolds were printed via printing needle (21G) based on a CAD model. The obtained scaffolds were dried at room temperature and then sintered at 1100 °C for 3h to obtain the final product [99]. The biological results demonstrated that this composite scaffold could induce more than 90% of in vitro MG-63 cell death and also inhibit tumor growth in an in vivo model. This scaffold material was able to induce osteogenic differentiation of MSCs derived from rabbits [99]. Recently, an innovative temperature-controlled 3D system, produced through glutaraldehyde and lyophilization for 2 days [100], and based on nano-hydroxyapatite/graphene oxide/chitosan (nHA/GO/CS) was described. The nHA/GO/CS system was able to eliminate osteosarcoma cells after near-infrared (NIR) irradiation (48 °C) and showed osteoinductive effects on MSCs at 42 °C by enhancing the BMP2/Smad signaling pathway [100]. In vivo tumor studies demonstrated that PTT is important for GO/CS and nHA/GO/CS to inhibit osteosarcoma cell growth. Moreover, an in vivo bone regeneration study showed that the best performance in terms of trabecular bone volume/total volume (c.a. 20%) was obtained for these scaffolds after 8 weeks of implantation [100].

Considerable attention has been devoted to black phosphorus (BP), which turned out to be a strong photocatalyst. BP has also proven to degrade to non-toxic products without bioaccumulation even for longer periods, another distinguished advantage making it stand out among the known two-dimensional (2D) materials. Recent studies have shown the use of an exfoliated BP-based nanosystem for cancer treatment. A novel and therapeutic BP-based scaffold was obtained by loading 2D BP in a three-dimensional (3D) bioglass scaffold. In particular, 2D BP was obtained by a liquid exfoliation approach by using N-methyl-2-pyrrolidone in a dark Ar glovebox. The BG scaffolds were obtained by 3D printing technique and then were soaked in 2D BP ethyl alcohol solution for 10 min and dried at room temperature. This procedure was repeated three times to obtain the final BG-2D BP scaffold [101]. The presence of 2D BP displayed important in vitro and in vivo photothermal properties for tumor ablation. Indeed, in vitro studies demonstrated that the scaffold with 2D BP supported the adhesion and proliferation of Saos-2 cells within 3 days of cell culture compared to a BG scaffold. However, lower viability of Saos-2 on BG-2D BP was observed than BG after PTT. In vivo studies confirmed that BG-2D BP shows multifunctional activities by reducing tumor growth and inducing new bone tissue formation in a physiological microenvironment [101]. Shao et al. [102] prepared an innovative system 2D BP-loaded thermosensitive hydrogel to be used after tumor removal surgery. The system is used as a sprayed hydrogel that is able to form a membrane on wounds under NIR irradiation and allowed the elimination of some residual tumor cells. Qin et al. [103] designed a thermosensitive hydrogel loaded with 2D BP and gemcitabine for photothermal and chemotherapy treatment. The encapsulation of 2D BP and gemcitabine was obtained by a “cold method”. In particular, Pluronic F-127 was dispersed in 2D BP solution and gemcitabine was added to the solution with magnetic stirring for overnight at 4°C to obtain a 2D BP/gemcitabine-loaded hydrogel.

The systems showed a fast sol-gel transition at physiological temperature upon NIR irradiation, good biodegradation, and photothermal stability. Furthermore, the hydrogel showed a good photothermal effect in Balb/c mice bearing 4T1 xenograft tumor. Indeed, chemotherapy using free gemcitabine and loaded hydrogel without PTT may only partially inhibit tumor growth. The best effects were obtained by combining PTT and chemotherapy.

However, Raucci et al. [104] investigated the role of 2D BP as anticancer agent for an experimental in vitro model of osteosarcoma without PTT treatment. 2D BP was obtained by liquid exfoliation of bulk BP in a DMSO solution and using ultrasounds process. Deoxygenated water was added to get a molar ratio P/H_2_O = 1–0.6.

The results demonstrated that 2D BP induces anti-proliferative and apoptotic effects by increasing the production of ROS on an osteosarcoma cell line (Saos-2), thus making 2D BP a powerful material in cancer therapy. Meanwhile, 2D BP shows an opposite behavior in the case of healthy human osteoblasts by inducing cell proliferation, osteogenic marker expression, and a protective effect against ROS [104].

**Figure 2:**
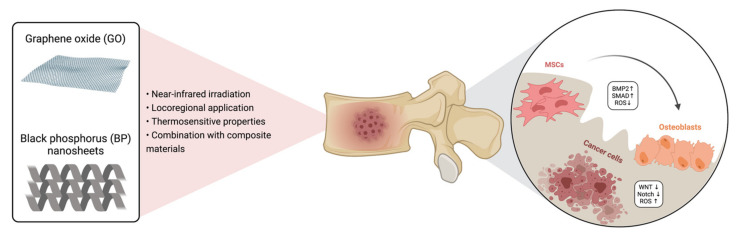
The use of GO and BP for the treatment of bone cancer. GO and BP may be delivered through a minimally invasive approach and activated in the tumor site under NIR irradiation. These materials have been shown to induce cancer cell apoptosis via increasing the production at the tumor niche but not in the healthy microenvironment and to reduce cell proliferation through the down-regulation of WNT and Notch pathways. Simultaneously, they stimulate MSC proliferation and differentiation toward mature osteoblasts, with an increase in ECM production. GO = graphene oxide; BP = black phosphorus; BMP = bone morphogenetic protein, SMAD = small mother against decapentaplegic; ROS = reactive oxygen species; MSCs = mesenchymal stem cells. Created with BioRender.com on 18 May 2021.

Moreover, in the last years, great attention has been paid toward quantum dots materials (i.e., phosphorus and carbon quantum dots) for osteosarcoma treatment. Indeed, the use of BP quantum dots (BPQDs) has been demonstrated by in vitro and in vivo tests as platforms for cancer treatment due to their ability to generate ROS [105] and as carriers of anticancer drugs for chemotherapy [106]. On the other hand, CDs are used as photothermal agents for bone tumors and infections. Lu et al. [107] reported the possibility to develop CS/hydroxyapatite scaffold-loaded CDs and demonstrated higher osteoinductive effects compared to a pure CS/hydroxyapatite scaffold both in vitro and in vivo. The scaffolds were produced by dissolving CDs at different concentrations in acetic acid, then hydroxyapatite nanoparticles and CS powders were added under constant stirring to obtain a final concentration of 1, 2 and 3 mg/mL. The mixture was frozen and freeze-dried in a lyophilized solution to obtain dried scaffolds. In an in vivo experiment, the scaffold with a CD at a concentration of 3 mg/mL showed significant photothermal properties with the highest reduction of tumor mass than other treatment groups under 1 W/cm^2^ NIR irradiation. Indeed, cells appeared necrotic with severe cell membrane damage [107]. Recently, the use of CD/WS_2_ material with an important photothermal effect in the NIR II range (100–1350 nm) was reported [108]. The CD/WS_2_ materials were synthesized by assembling CDs and WS_2_ nanosheets under magnetic stirring at room temperature in a PBS solution. The obtained system was dispersed in water at different concentrations for biological investigations. In particular, for this system, a deep-tissue penetration with complete ablation of MG-63 tumor mass was reported after 18 days of intravenous injection treatment and under 1064 nm laser irradiation (0.6 W/cm^2^), in the presence of additional tissue (10 mm-thickness). Furthermore, the system was able to induce in vitro and in vivo osteogenic differentiation with higher expression of heat shock protein-70 (Hsp70), demonstrating the ability to repair and regenerate bone defect by hyperthermia treatment. The main characteristics of innovative and multifunctional biomaterials for the treatment of bone cancer are summarized in Figure 2. A summary of reviewed studies is depicted in Table 3.

## 4. Current Perspective and Future Orientations

The treatment of bone cancer poses a great challenge to contemporary medicine, due to both its significant frequency and the high heterogeneity of malignant lesions within the bone tissue. Traditional treatments, either surgical or conservative, are rarely curative and usually consent to only temporarily ameliorate patients’ quality of life and survival [109]. Furthermore, these approaches fail to restore bone original morpho-functional properties and are often aggravated by serious adverse effects and irreversible damage to the bone tissue. Indeed, bone is a natural composite material lacking an artificial correspondent and thus being the second tissue to require transplantation after blood [109,110]. Treatment protocols for bone cancer strictly depend upon the primary or secondary nature of the lesion [111,112]. Generally, the existence of a bone metastasis is indicative of an advanced-stage primary tumor, against which available treatments are infrequently resolutive. In these cases, applicable strategies aim to prolong patient survival and ameliorate their quality of life through the prevention of SREs [109]. Current pharmacologic approaches include anti-resorptive drugs such as bisphosphonates and denosumab (an anti-RANK-RANKL inhibitor), which inhibit osteoclast activity thus reducing cancer-related pain, fractures, and the risk of developing new lesions [113]. However, cancer recurrence and progression in patients already on these treatments has encouraged the exploration of advanced targeted therapies such as anti-cathepsin K (e.g., odanacatib) and anti-sclerostin (e.g., romosuzumab) inhibitors. Notwithstanding, the former is no longer under development due to increased risks of stroke and other adverse events [114], while the latter has been recently approved by the FDA for the treatment of postmenopausal osteoporosis, with specific warnings for increased cardiovascular events [115].

As far as osteosarcoma is concerned, standard care currently consists of neoadjuvant chemotherapy based on the administration of DOX, CDDP and high-dose MTX, followed by surgical resection [116]. In both primary and secondary bone cancer treatment, surgery should have the goal to remove the tumor while preserving as much function as possible; in this regard, bone replacement and/or the use of prostheses and bioimplants may significantly reduce the disability burden [109]. Autologous bone grafting remains the gold standard due to its optimal biocompatibility, no risk of immune response and relevant regenerative potential; however, the restricted availability of tissues and donor-site morbidity are often a considerable concern [117]. On the other hand, allografts and xenografts may overcome some of these limitations but are burdened by high costs, poor compatibility with host tissues and risk of rejection or infection [109]. Therefore, tissue engineering approaches are being developed to fill such an existing gap through the design of biocompatible, structurally valid, and multifunctional materials that may foster bone regeneration and anticancer effects at the same time. To date, fourth generation biomaterials offer the possibility to develop smart biodevices with both anti-tumor and anti-infective properties which are able to achieve a controlled release of bioactive molecules and the activation of specific pathways upon distinct signals from the surrounding microenvironment [109]. In addition, such devices are often applied through loco-regional approaches, which reduce the risk of side effects derived from the systemic administration of antineoplastic drugs while achieving a high local drug concentration with minimal invasiveness [118]. In the last decade, several nanocarrier-based drug delivery systems have been described. These include organic, inorganic and hybrid materials provided with enhanced pharmacokinetics, reduced toxicity, and sustained pharmacologic release compared to conventional treatments [119]. Whilst organic nanocarriers alone represent valid drug carriers, they are characterized by poor mechanical properties and a high degree of degradation following administration. However, innovative hybrid materials have been proven to retain their drug delivery features while presenting adequate biomechanical characteristics for bone implantation and loco-regional administration [120]. For instance, the application of these biomaterials through minimally invasive approaches often requires tuning their physicochemical mechanical properties in order to consent their use as injectable systems [121]. Nonetheless, the development of these devices is affected by significant costs and the need for optimizing fabrication processes, drug release kinetics and structural features is essential to promote their application in the bigger clinical scenario [109]. The needs of drug therapy still have some aspects to be investigated in terms of matrix stability, cell membrane crossing, and the design of multiple drug delivery nanosystems with a controlled kinetics release. From a manufacturing point of view, 3D printing technology has an important role in the development of 3D-printed drug delivery systems for personalized drug-loaded medical devices and dosage forms improving and providing various possibilities for meeting the needs of personalized drug therapy. However, a great effort should still be done to design and develop drug delivery systems able to overcome some issues that are still remaining such as permeability, bioavailability and retention effects of drugs in tumor site. Thus, future research should be addressed to enhance the permeation and retention (EPR) effect and oriented toward the development of multifunctional, tunable materials that may be able to specifically target cancer cells while respecting the healthy tissue microenvironment, and that may be developed and delivered according to a tailored approach based on the type of tumor and patients’ unique characteristics.

## Figures and Tables

**Table 1:** Summary of studies on inorganic drug delivery systems for the treatment of bone cancer.

Primary Material	Study Type	System Composition	Experimental Model	Ref.
CaCO_3_	In vitro/in vivo	HA-DOX/CaCO_3_	Mouse osteosarcoma K7 cells—osteosarcoma-allografted BALB/c mouse model	[63]
	In vitro/in vivo	^Ca^NP/DOX	Mouse osteosarcoma K7 cells/human osteosarcoma 143B cells—orthotopic osteosarcoma BALB/c mouse model	[66]
CeO_2_	In vitro/in vivo	PPCNPs-Ce6/FA	MCF-7 and MCF-7/ADR cells—xenografted BALB/c mouse model	[67]
	In vitro	Dextran-coated nanoceria	Human osteosarcoma MG-63 cells	[68]
	In vitro	Na_2_CO_3_ + CeO_2_ nanoparticles + DOX + COL I	Human osteosarcoma Saos-2 cells	[69]
MS	In vitro	AMBGs + AL	Human osteosarcoma MG-63 cells	[71]
	In vitro/in vivo	Au@MSNs-ZOL	Human MDA-MB231 cells—xenografted NU/NU mouse model	[72]
	In vitro	DOX + MS nanoparticles + PAA + ConA	Human osteosarcoma HOS cells	[73]
CaP	In vitro/in vivo	CPPA/DOX	Human osteosarcoma 143B/MG63/U2Os/Saos-2 cells—xenografted BALB/c mouse model	[78]
	In vitro	DOX-loaded CaP porous microspheres	Human osteosarcoma 143B/MG63 cells	[79]

AL = alendronate; AMBGs = aminated mesoporous bioactive glass; Au@MSNs-ZOL = gold nanorods enclosed inside MS nanoparticles conjugated with ZOL; CaCO_3_ = calcium carbonate; CaNP/DOX = (CaCO_3_)-core-crosslinked nanoparticle of methoxy poly(ethylene glycol)-block-poly(l-glutamic acid) through mineralization for intracellular delivery of doxorubicin; CaP = calcium phosphate; CeO_2_ = cerium oxide; COL I = collagen type I; ConA = concanavalin A; DOX = doxorubicin; CPPA = calcium phosphate-phosphorylated adenosine; HA = hyaluronic acid; MS = mesoporous silica; PAA = polyacrylic acid; PPCNPs-Ce6/FA = chlorin e6/folic acid-loaded branched polyethylenimine-PEGylation ceria nanoparticles; ZOL = zolendronic acid.

**Table 2:** Summary of studies on polymeric drug delivery systems for the treatment of bone cancer.

Primary Material	Study Type	System Composition	Experimental Model	Ref.
CS	In vitro/in vivo	Chi/DPO + pPEDF	Human osteosarcoma Saos-2 cells—orthotopic osteosarcoma BALB/c mouse model	[81]
	In vitro	CuCNPs	Human osteosarcoma MG-63 cells	[85]
	In vitro	HMWC/LMWC	Human osteosarcoma Saos-2 cells	[86]
	In vitro	CS-sodium alginate + TiO_2_ nanoparticles	Human osteosarcoma MG-63 cells	[87]
	In vitro/in vivo	ZSM-5/CS/DOX	Human osteosarcoma MG-63 cells/Sprague-Dawley rat	[88]
PLGA−PEG− PLGA	In vitro/ex vivo/in vivo	PLGA−PEG− PLGA + DOX/CDDP/MTX	Human osteosarcoma MG-63/Saos-2 cells—orthotopic osteosarcoma BALB/c mouse model	[82]
PGA	In vitro	MTX-PGA	Human osteosarcoma 791T/Saos-2 cells	[83]
HA	In vitro/in vivo	ALN−HA−SS−L functionalized liposomes	Human osteosarcoma MG63 cells—orthotopic osteosarcoma BALB/c mouse model	[89]
	In vitro/in vivo	^CDDP^HANG/DOX	Mouse osteosarcoma K7 cells—xenografted BALB/c mouse model	[91]
	In vitro/in vivo	HA-PEG-nHA-ZOL NP	293T cells—C57BL/6 mice	[92]
	In vitro/in vivo	^CDDP^NP_DOX&R848_	Mouse osteosarcoma K7M2 cells—orthotopic osteosarcoma BALB/c mouse model	[93]

ALN−HA−SS−L = alendronate-hyaluronic acid-disulfide bond-lipid; CDDP = cisplatin; ^CDDP^HANG/DOX = cisplatin-crosslinked hyaluronic acid nanogel load with doxorubicin; ^CDDP^NP_DOX&R848_ = hyaluronic acid nanoparticle loaded with cisplatin, doxorubicin and resiquimod; Chi/DPO = chitosan/dipotassium orthophosphate; CS = chitosan; CuCNPs = copper-loaded chitosan nanoparticles; DOX = doxorubicin; HA-PEG-nHA-ZOL NP = hyaluronic acid-poly(ethylene glycol)-nano-hydroxyapatite-zoledronic acid nanoparticles; HMWC = high molecular weight chitosan; LMWC = low molecular weight chitosan; MTX = methotrexate; pPEDF = pigment epithelium-derived factor plasmid; PEG = poly(ethylene glycol); PGA = poly(glycerol adipate; PLGA = poly(L-lactide-co-glycolide).

**Table 3:** Summary of studies on innovative multifunctional biomaterials for the treatment of bone cancer.

Primary Material	Study Type	System Composition	Experimental Model	Ref.
GO	In vitro	GO-TCP	Human osteosarcoma MG-63 cells/rabbit BMSCs	[99]
	In vitro/in vivo	nHA/GO/CS	Human osteosarcoma HOS cells/MC3T3-E1 cells/human BMSCs—xenografted BALB/c mouse model	[100]
BP	In vitro/in vivo	BP-BG	Human osteosarcoma Saos-2 cells—orthotopic osteosarcoma BALB/c mouse model	[101]
	In vitro/in vivo	BP nanosheets + gemcitabine	Human 4T1 cells—xenografted BALB/c mouse model	[103]
	In vitro	2D BP	HOb/hMSCs/human osteosarcoma Saos-2 cells	[104]
CD	In vitro/in vivo	CS/nHA/CD	Rat osteosarcoma UMR106 cell/rabbit BMSCs—xenografted BALB/c mouse model	[107]
	In vitro/in vivo	CD/WS_2_ HJs	Human osteosarcoma MG-63 cells—xenografted BALB/c mouse model	[108]

2D = two-dimensional; BP = black phosphorus; BP-BG = black phosphorus bioglass; CD = carbon dots; CS/nHA/CD = chitosan/nanohydroxyapatite carbon dots; GO = graphene oxide; GO-TCP = graphene oxide-modified β-tricalcium phosphate; hMSCs = human mesenchymal stem cells; HOb = human preosteoblasts; nHA/GO/CS = nano-hydroxyapatite/graphene oxide/chitosan; BMSCs = bone mesenchymal stem cells.

## Data Availability

Not applicable.
